# Supplementary material for: Bladder Cancer Metastasis Induced by Chronic Everolimus Application Can Be Counteracted by Sulforaphane In Vitro
Source: Int J Mol Sci. 2020 Aug 4;21(15):5582. doi: 10.3390/ijms21155582 (PMC7432076; doi:10.3390/ijms21155582)
Supplement: Supplementary file 1 [file ijms-21-05582-s001.zip › ijms-833377-supplementary.pptx]

## Slide 1
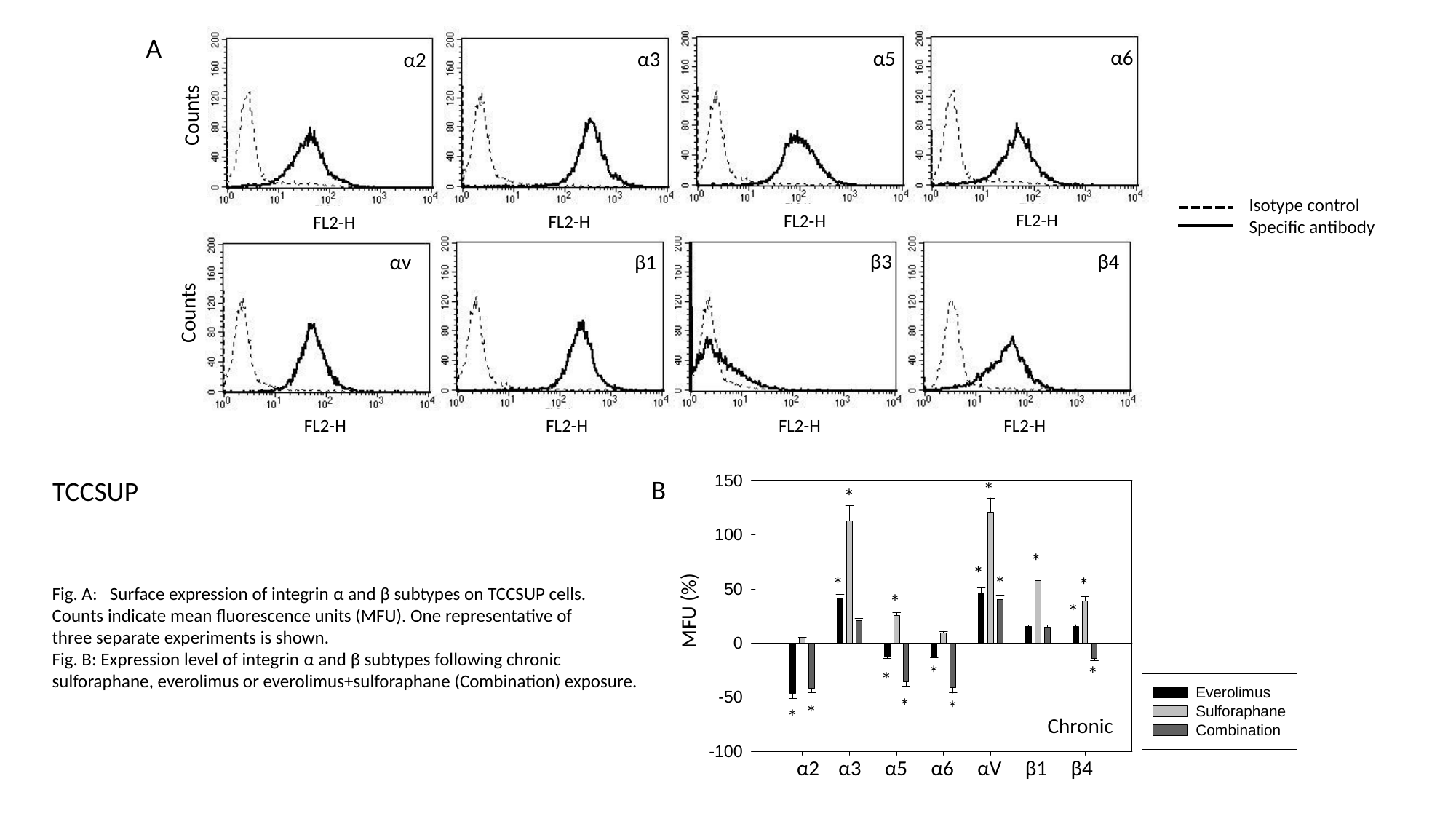

A
α6
α5
α3
α2
Counts
Isotype control
Specific antibody
FL2-H
FL2-H
FL2-H
FL2-H
β4
β3
αv
β1
Counts
FL2-H
FL2-H
FL2-H
FL2-H
B
TCCSUP
*
*
*
*
*
*
*
Fig. A: Surface expression of integrin α and β subtypes on TCCSUP cells.
Counts indicate mean fluorescence units (MFU). One representative of
three separate experiments is shown.
Fig. B: Expression level of integrin α and β subtypes following chronic
sulforaphane, everolimus or everolimus+sulforaphane (Combination) exposure.
*
MFU (%)
*
*
*
*
*
*
*
*
Chronic
α2 α3 α5 α6 αV β1 β4

## Slide 2
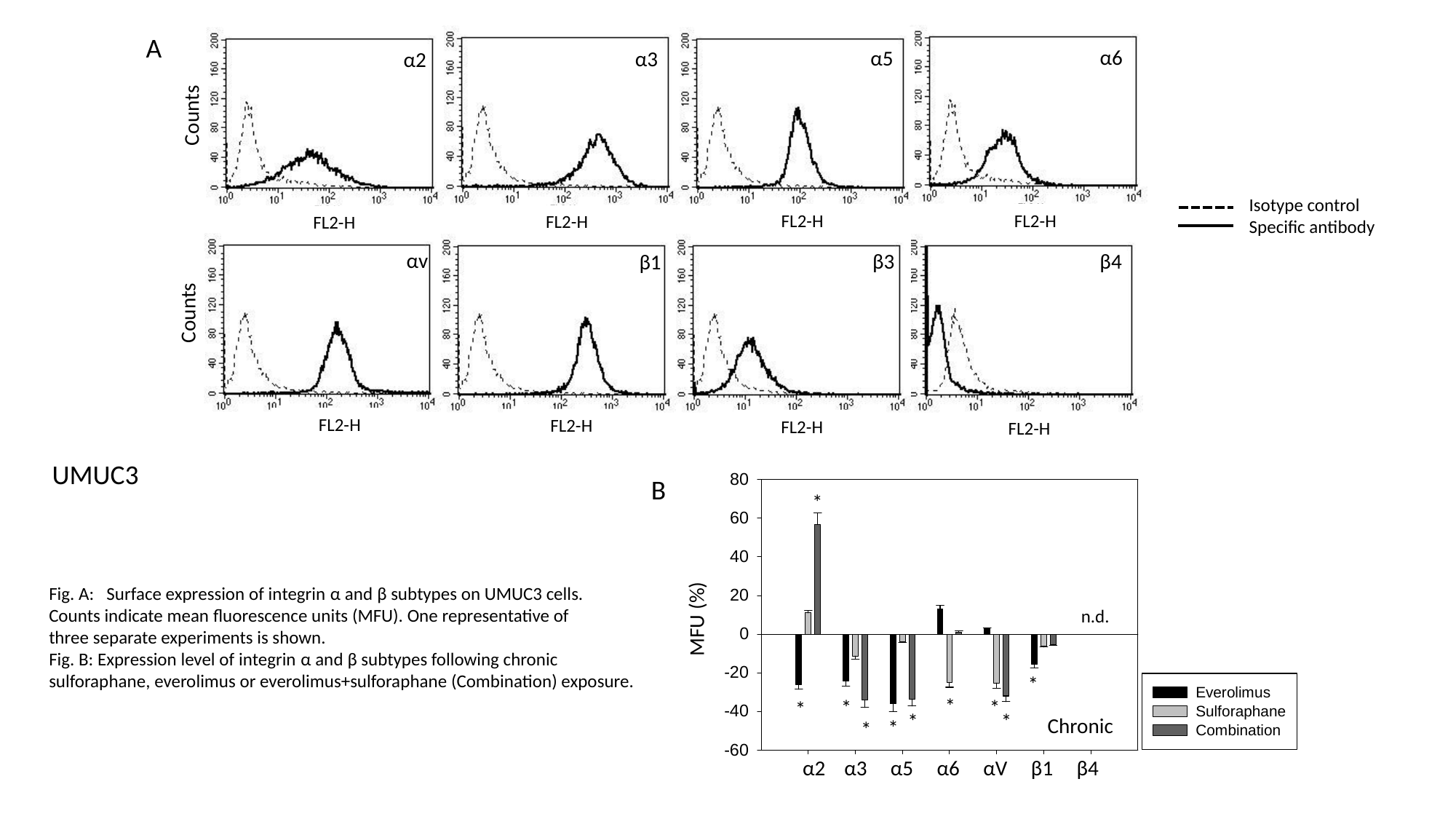

A
α6
α5
α3
α2
Counts
Isotype control
Specific antibody
FL2-H
FL2-H
FL2-H
FL2-H
αv
β4
β3
β1
Counts
FL2-H
FL2-H
FL2-H
FL2-H
UMUC3
B
*
Fig. A: Surface expression of integrin α and β subtypes on UMUC3 cells.
Counts indicate mean fluorescence units (MFU). One representative of
three separate experiments is shown.
Fig. B: Expression level of integrin α and β subtypes following chronic
sulforaphane, everolimus or everolimus+sulforaphane (Combination) exposure.
n.d.
MFU (%)
*
*
*
*
*
*
*
Chronic
*
*
α2 α3 α5 α6 αV β1 β4

## Slide 3
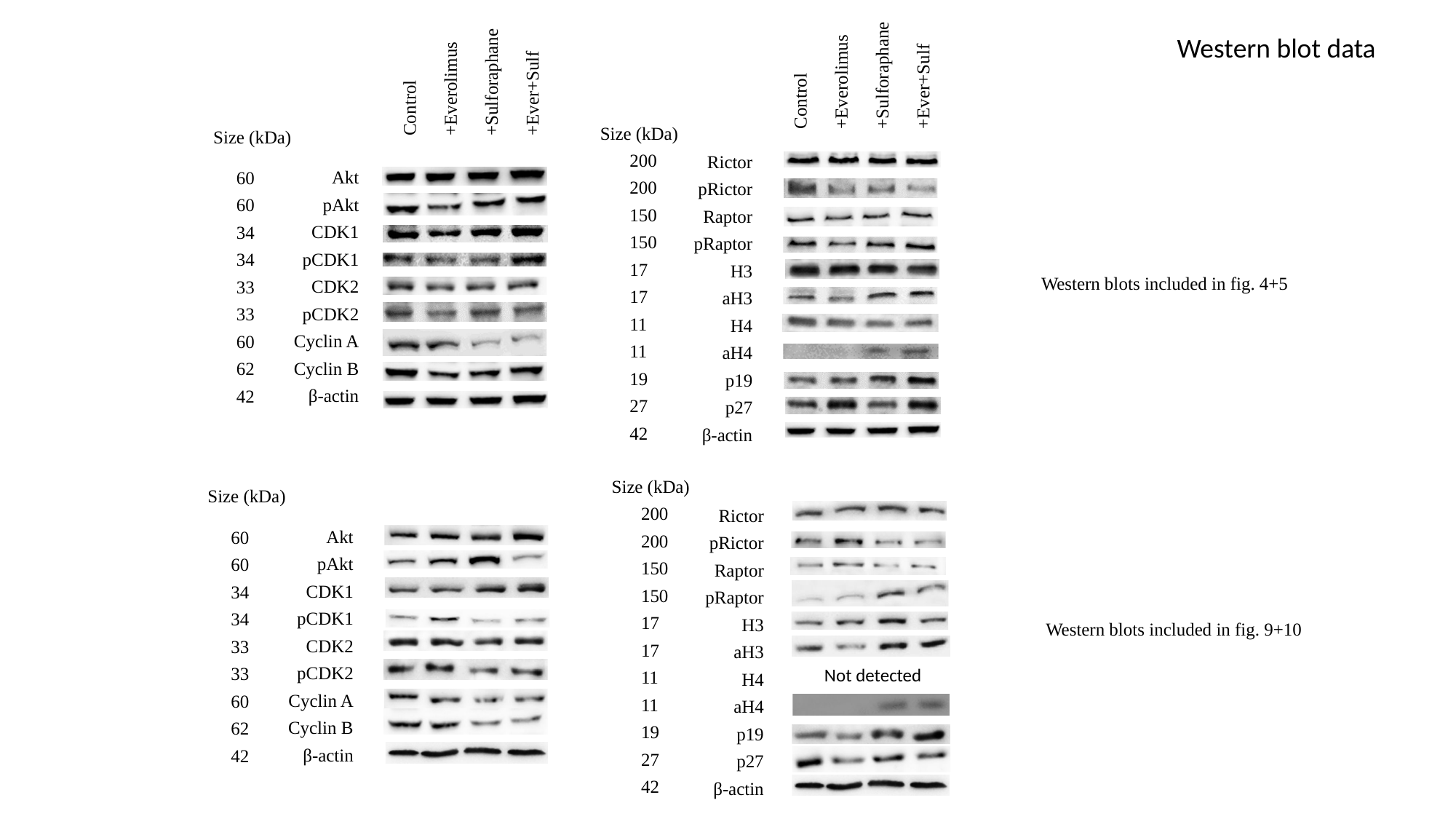

Control
+Everolimus
+Sulforaphane
+Ever+Sulf
Control
+Everolimus
+Sulforaphane
+Ever+Sulf
Western blot data
Size (kDa)
Size (kDa)
200
200
150
150
17
17
11
11
19
27
42
Rictor
pRictor
Raptor
pRaptor
H3
aH3
H4
aH4
p19
p27
β-actin
Akt
pAkt
CDK1
pCDK1
CDK2
pCDK2
Cyclin A
Cyclin B
β-actin
60
60
34
34
33
33
60
62
42
Western blots included in fig. 4+5
Size (kDa)
Size (kDa)
200
200
150
150
17
17
11
11
19
27
42
Rictor
pRictor
Raptor
pRaptor
H3
aH3
H4
aH4
p19
p27
β-actin
Akt
pAkt
CDK1
pCDK1
CDK2
pCDK2
Cyclin A
Cyclin B
β-actin
60
60
34
34
33
33
60
62
42
Western blots included in fig. 9+10
Not detected
